# Construction and Identification of a Recombinant Plasmid Encoding *Echinococcus granulosus* Oncosphere Antigen (EG95)

**Published:** 2017

**Authors:** Nahideh MAZAHERI, Abdolhossein DALIMI, Majid PIRESTANI, Farnoosh JAMEIE, Mehdi MOHEBALI, Mohamad Bagher ROKNI

**Affiliations:** 1. Dept. of Medical Parasitology and Mycology, School of Public Health, Tehran University of Medical Sciences, Tehran, Iran; 2. Dept. of Parasitology, Faculty of Medical Sciences, Tarbiat Modares University, Tehran, Iran; 3. Center for Research of Endemic Parasites of Iran (CREPI), Tehran University of Medical Sciences, Tehran, Iran

**Keywords:** *Recombinant plasmid*, *Oncosphere*, *EG95*, *Echinococcus granulosus*

## Abstract

**Background:**

Cystic echinococcosis (CE), as a zoonotic disease cause to health threat and economic losses. Despite implemented control programs, few countries have been able to decrease or eliminate this infection. Vaccination of the intermediate host offers an additional strategy to control the parasite transmission and EG95 antigen is considered more than the others in the vaccine issue. According to the high protection induced by the EG95 recombinant vaccine, this study was designed to construct recombinant plasmid formulation of EG95 antigen.

**Methods:**

In 2015, the *Echinococcus granulosus* eggs were recovered from an infected dog in Parasitological laboratory of Tarbiat Modares University in Tehran, Iran. Following hatching, the oncospheres of *E. granulosus* were activated to increase the presence of the desired mRNA. The extracted mRNA was transcribed to the cDNA which used as template in RTPCR. Then the EG95 gene cloned into pET28a vector and the recombinant plasmids expression was investigated in prokaryotic and eukaryotic cells.

**Results:**

The recombinant plasmid encoding EG95 antigen was successfully constructed and identified by PCR, restriction enzyme digestion and sequencing. In vitro expression of the EG95 antigen was confirmed in prokaryotic and eukaryotic systems by SDS-PAGE and western blotting analysis.

**Conclusion:**

Because of potential advantages of DNA vaccines, including ability to induce long-term immune responses, low production cost and stability in different temperatures, this study carried out to construct the EG95 gene into a vector. This recombinant vector can be evaluated in further studies as a DNA vaccine may provide new prospects for the development of a vaccine against cystic hydatid disease.

## Introduction

Cystic echinococcosis (CE), as an important and neglected public health problem, caused by larval stage of canine tapeworm *Echinococcus granulosus sensu lato* (1, 2). Involvement of vital organs such as the liver and lungs in CE results in considerable health threat and economic losses, particularly in regions where pastoral activities are common (2–4). Infection by this parasite has widespread distribution and has been reported from Central Europe, Central Asia, Mediterranean countries, the Far East, the Middle East, South America, East Africa and Australia (2). Iran is one of the endemic areas (5).

Despite implemented control programs around the world, few countries have been able significantly to decrease or eliminate this infection through the general education and dog control (3). Vaccines offer an additional strategy to control the parasite transmission (3). The parasite has a wide range of antigenic proteins and because of its high host-protective potential against infection; EG95 is considered more than the others in the vaccine issue (6). EG95 recombinant vaccine was developed in 1996 which induced high protection (96–98%) in sheep and vaccine trials carried out in Australia, Argentina and China have confirmed the efficacy of the vaccine (7–9).

Due to complex production and purification process of recombinant vaccines, specific knowledge and equipment are required. In addition, because of protein structure of these vaccines cold chain storage and transportation are needed cause to increase in the cost of vaccine production. Moreover recombinant vaccines like exogenous antigens and killed vaccines only stimulate humoral immune response, whereby DNA vaccines generate cellular, humoral and mucosal immunity. Immunization against different strains, need to fewer amount of vaccine, multivalent vaccines and inducing long-lasting immune responses could be considered as potential advantages of DNA vaccines which can be referred. Stability at different temperatures and cost-effectiveness are the most important advantages of DNA vaccines (10).

One rationale for efforts to develop DNA vaccine against *Echinococcosis* is that cost is an important factor for vaccines to be administered in livestock industries (3). Therefore, considering the high immunogenicity of EG95 recombinant vaccine and high production cost of these vaccines, this study was conducted to construct a plasmid encoding EG95 antigen.

## Materials and Methods

### Collection, hatching and activation of oncospheres

A 6-month-old male dog had been previously infected by feeding with protoscoleces of sheep origin. Two months after infection, the dog was euthanized and the adult worms were collected from the small intestine. In order to recover the eggs from the gravid proglottids, the worms were passed through the filter and suspended in normal saline according to the required number of eggs. The eggs were poured in 10% sodium hypochlorite resulted in the eggshell crack. When this step was followed by exposure to a solution containing artificial intestinal fluid (AIF) solution (pancreatin, Na-HCO3 and sheep bile) and incubation at 37 °C for 1 hour, the viable oncospheres were activated and left the oncospheral membrane (6). The activated oncospheres were stored immediately in liquid nitrogen until used for RNA extraction.

The study was approved by Ethics Committee of Tehran University of Medical Sciences, Tehran, Iran.

### Construction of plasmid encoding EG95 and preparation

The total RNA was extracted from the oncosphere using TRIzol reagent followed by quantification using a spectrophotometer (Nano Drop) at wavelengths of 230, 260, and 280 nm. A transcription kit, the *AccuPower*® *CycleScript RT PreMix*, k-2046 (BIONEER), was applied to amplify all mRNA into complementary DNA (cDNA) according to the manufacturer’s instruction (11). The open reading frame of *E. granulosus* EG95 was amplified for 35 cycles (an initial denaturation at 94°C for 5 min, followed by 35 cycles of 30 s at 94°C, 30 s at 53°C, 30 s at 72°C and final extension at 72°C for 10 min). PCR amplification was performed using the following primers in which restriction sites were inserted as underlined below. F: 5′-CGGAATTCATGGCATTCCAGTTATGTCTC-3′ (*EcoRI*) and R: 5′-GCCTCGAGTCAAGTAAGGACAAC-3′ (*XhoI*) (12).

The PCR product was ligated into the pET28a (+) vector (Novagen) by T_4_ DNA ligase (Thermo scientific) at the *EcoRI* and *XhoI* sites to construct recombinant plasmid pET28a/His-EG95. *E.coli* Top10 was transformed with the resultant recombinant plasmid. The insert was purified by a mini columns plasmid purification kit (GeneAll, Korea) and confirmed by restriction enzyme digestion, PCR and sequencing.

### Expression of EG95 protein in bacteria

The recombinant histidine tagged pET28a-EG95 expression plasmid was transformed to *E.coli* BL21 (DE3) pLysS competence cells with kanamycin selection (25μg/ml). The recombinant protein was harvested after 4 hours of induction with 1.0 mM IPTG (isopropyl b-D-1-Thiogalactopyranoside). Protein expression in *E.coli* BL21 (DE3) pLysS was confirmed by western blotting. In brief, cells were harvested after induction, disrupted by sonication and proteins in the supernatant of the lysate were separated by SDS-PAGE and blotted onto a nitrocellulose membrane, which was then blocked with 2% BSA (bovine serum albumin). The nitrocellulose membrane was incubated with HRP (horseradish peroxidase)-labeled murine anti-His antibodies (Agrisera) diluted 1:1000 in TBST (Tris Buffered Saline containing Tween 20; 20 mM Tris–Cl pH: 7.8, 0.5 M NaCl, 0.5% Tween 20). Then the enzyme provided with 3, 3′-Diaminobenzidine (DAB) as a substrate that readily converted to its oxidized form, forming a brown precipitate on the membrane.

### PEG95 plasmid expression in Human Embryonic Kidney Cell-line

#### 293T (HEK293T) Cells

The recombinant plasmid encoding EG95 (pcDNA3.1-EG95) was used for expressing the EG95 protein in HEK293T cells. HEK cells were routinely cultured in Dulbecco’s modified Eagle’s medium (DMEM-F12) supplemented with streptomycin (100 mg/ml) and penicillin (100 IU/ml), 10% fetal bovine serum (FBS) at 37°C with 5% CO2. Cell density should be 50–80% confluent on the day of transfection. Briefly, before transfection the cells were seeded in a 24-well plate. The recombinant pcDNA3.1-EG95 plasmid (1μg/well) was transfected into cells using lipofectamine 2000 reagent (Invitrogen) according to the manufacturer’s instructions (13). Enhanced green fluorescent protein (EFGP) expression in transfected HEK cells was used as control. The recombinant plasmid encoding EGFP (pEGFP) was transfected into HEK cells by lipofectamine.

The transfected and untransfected cells were used as the positive and negative controls respectively (14). Plates were incubated for 48 h at 37°C in a humidified atmosphere of 5% CO2. Forty-eight hours after transfection, EGFP expression was determined using fluorescence microscopy. According to the screening for the G418 resistance marker gene of the pcDNA3.1 vector, positive cells were scraped from plate and lysate of cells were separated by 12% sodium dodecyl sulfatepolyacrylamide gel (SDS–PAGE). The EG95 related gene expression was detected in the cells by western blotting analysis as previously described (14, 15).

## Results

### Construction of recombinant plasmid pET28a-EG95

**Fig.1:**
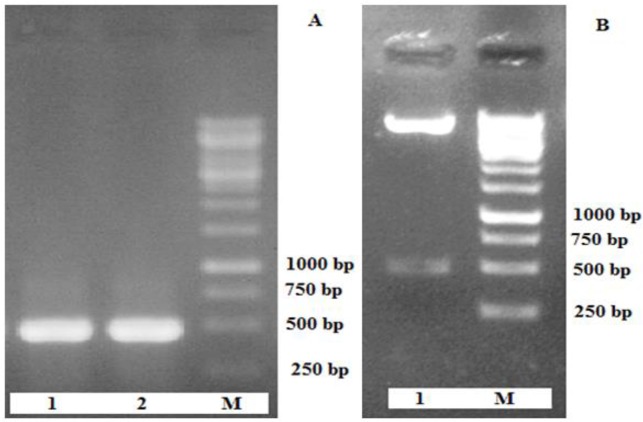
The construction of DNA vaccines expressing EG95antigen. The recombinant plasmid pcDNA3.1/EG95 (A) were identified by PCR amplification (Lane 1, 2) and (B) restriction enzyme digestion analysis (Lane 1). Lane M: DNA marker

Total RNA was extracted from activated oncospheres of *E. granulosus*, and reverse-transcribed into cDNA. Then this cDNA were used as templates to conduct RT-PCR. The PCR products were analyzed using 1% agarose gel electrophoresis, and specific bands showed at 471 bp, which was consistent with the expected result. The recombinant plasmid pET28a-EG95 recombinant plasmid was constructed and identified by PCR (Fig. 1. A), and by digestion with *EcoRI* and *XhoI* restriction enzyme (Fig. 1. B). A fragment of 471 bp was obtained through either PCR or digestion methods. The recombinant plasmid further confirmed by sequencing analysis.

### Sequencing analysis

The digested recombinant plasmid was sent for sequencing. The comparison of the recorded EG95 antigen-specific sequence in GenBank (accession no. KY661711) showed that the cloned EG95 antigen gene sequence was also consistent with the Eg95 antigen gene sequence in GenBank.

### In vitro expression of EG95 gene in prokaryotic cells

**Fig. 2:**
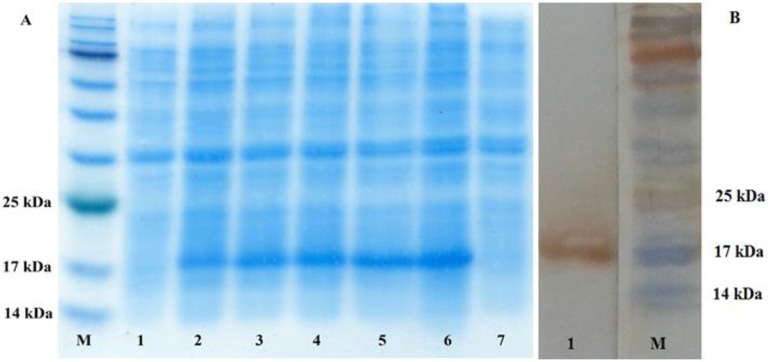
Identification of EG95 expression in *E.coli* BL21 (DE3) pLysS bacteria by (A) SDS-PAGE and (B) western-blotting. M: prestained protein marker. A: lane 1: Non-induced bacteria transfected with pET28a-*EG95*; 2: 1-hour induced bacteria transfected with pET28a-*EG95* with 1 mmol/L IPTG; 3: 2-hour induced bacteria transfected with pET28a-*EG95*; 4: 3-hour induced bacteria transfected with pET28a-*EG95*; 5: 4-hour induced bacteria transfected with pET28a-*EG95*; 6: 5-hour induced bacteria transfected with pET28a-*EG95;* 7: Bacteria transfected with empty pET28. B: Lane 1: rEG95-His/pET28a detected with the anti-His monoclonal antibodies

The expression of His-EG95 was investigated in prokaryotic and eukaryotic systems. The molecular weight of the recombinant protein encoded by 156 amino acids was about 17 kDa. To express His-EG95 gene in vitro, the prokaryotic expression vector pET28a/His-EG95 was induced for expression by IPTG. After separation by SDS-PAGE there was an obvious band at 17 kDa after induction. As shown (Fig. 2.A) expression was apparent in *E.coli* BL21 (DE3) pLysS transfected with pET28a /His-EG95, but not in those control cells which were transfected with the empty vectors.

The results demonstrating the recombinant protein His-EG95 was successfully expressed in *E.coli* BL21 (DE3) pLysS and the protein amount were gradually increased with prolonged induction. To verify the expression of His-EG95 protein, Western blotting analysis was conducted. The result showed that the anti-His-tag antibody could recognize the protein at the position of approximately 17 kDa. No band was found when the antibody reacted with the control cells which were transfected with the empty plasmids (Fig. 2. B).

### In vitro expression of EG95 gene in eukaryotic cells

**Fig.3:**
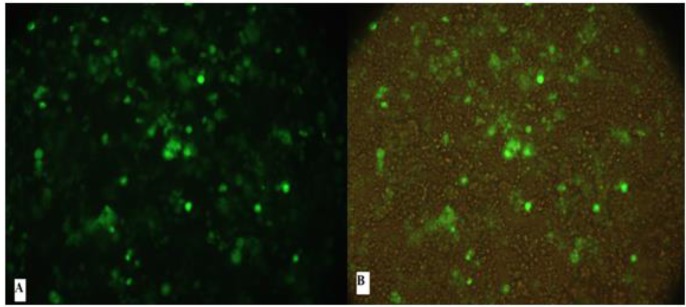
Detection of the in vitro expression of the recombinant plasmid pcDNA3.1/His-EG95 in transfected HEK293T cells by fluorescence microscopy. (A, B) HEK293 cells were transfected with pEGFP-N1at 48 h post-transfection

To check the expression of EG95 in the eukaryotic cell, plasmid pcDNA3.1/His-EG95, was constructed and transfected into HEK293T cells. Forty-eight hours after transfection, EGFP expression was determined in the cells transfected with pEGFP (as positive control) using fluorescence microscopy (Fig. 3). Furthermore the level of protein expression was confirmed by Western blotting.

**Fig. 4:**
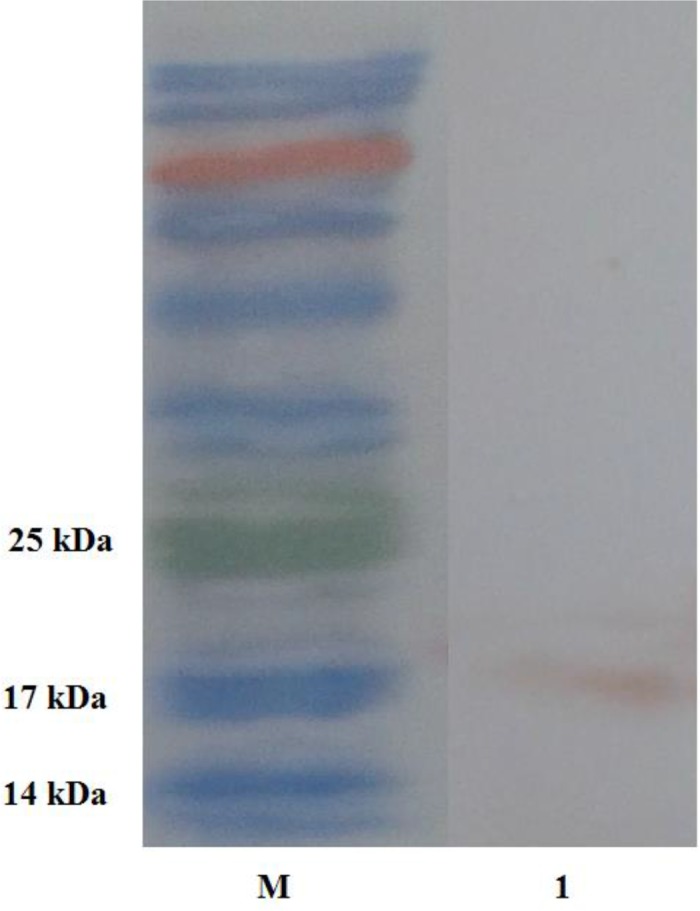
Identification of EG95 expression in HEK293T cells by western-blotting. M: Prestained protein marker; Lane 1: rEG95-His/pcDNA3.1 detected with the anti-His monoclonal antibodies

Electrophoresis of HEK293T transfected with pcDNA3.1/His-EG95 showed one band of about 17 kDa, identified by Western blotting using anti-His monoclonal antibodies. The cells transfected with the empty pcDNA3.1 showed no similar band (Fig. 4).

## Discussion

DNA vaccines as a type of subunit vaccine offer promise to the development of needed vaccines and the improvement of existing vaccines. In contrast to most protein subunits, DNA vaccines induce long-term humoral and cellular immunity. This is due to expression of proteins within host cells that allow to access pathways for presentation by both class I and class II MHC (major histocompatibility antigens) (16). In vivo protein synthesis allows the processing and presentation of the protein to the host’s immune system in a way similar to that which would arise during a natural infection (17) whereas; many factors affect the practicality of recombinant vaccine proteins. Theoretically, the proteins synthesized through expression of recombinant clones are identical. While this is true with consideration the amino acid sequence of expressed protein, it does not necessarily represent the quality of the practical product. Various expression and processing conditions may impinge on the proteins secondary structure causing significant changes in solubility and immunogenic characteristics of proteins in recombinant vaccines (3).

Cystic Echinococcosis is a cosmopolitan zoonosis with substantial socio-economic consequences (1). Vaccination of the intermediate hosts is a potential strategy to improve the effectiveness of disease control programs. A considerable amount of research has been undertaken on various aspects of EG95 as a vaccine candidate. The recombinant EG95 protein vaccine has now been demonstrated effective in conducted vaccine trials in Australia, Argentina and China (7–9, 18). In his observations on antibody responses in sheep immunized with EG95 as a fusion protein with glutathione S-transferase (GST), Woollard indicated that the protective effects of the EG95 vaccine associated with the specific antibody to the linear immunogenic regions of EG95 (19). According to these findings and other studies implied on the immunogenic function of EG95 (20–22), this antigen was selected to construct a recombinant plasmid as a vaccine in the current study.

The substantial evidences indicated that oncospheres contain host-protective proteins (23). In mature oncospheres Heath and Lawrence identified the antigenic polypeptides involved in the protective immunity to *E. granulosus* infection. The results indicated the presence of protective epitopes in these antigens (24). In a histological study carried out to determine the localization of EG95 antigen, the oncospheral penetration glands were found to be the particularly rich source of EG95 antigens (6). Following activation these antigens are secreted by oncospheres. Therefore, we used the oncosphere as the source of antigen and prior to extraction of mRNA the oncospheres of *E. granulosus* were activated to increase the presence of the desired mRNA. This mRNA was transcribed to cDNA of the EG95 resulting to the cloning of the EG95 antigen.

Chow illustrated a gene family of seven members (EG95_1–7_) is related to expression of EG95 protein and four subunits (EG95 _1–4_) expressing an identical protein had been used by Lightowlers in recombinant EG95 vaccine (25, 26). These four subunits which was also used in our study, have transcription confined to the oncosphere. Whereas, the other two members (EG95 _5, 6_) which express a variant protein were transcribed in the all life cycle stages (26) and in their studies Sarvi (12) and Lin (27) constructed a recombinant plasmid encoding these subunits of native EG95 gene family with protoscoleces origin.

Advantages of the use of direct DNA inoculations for protein expression, including induction of long-lasting immune responses, low production cost and stability in different temperature, and immunogenic role of EG95 protein led us to construct an EG95-expressing vector DNA. The EG95 gene was successfully cloned in to pET28a and the result of sequencing indicated that it is different from the EG95 isolated in Sarvi′ s study and was 492 bp (28). The pET28a-EG95 recombinant plasmid was expressed in prokaryotic and eukaryotic cells.

## Conclusion

EG95 gene was constructed into a vector to be evaluated in further studies. This may provide new prospects for the development of a DNA vaccine against cystic hydatid disease.
